# Robust antibody and T cell responses tracked longitudinally in patients with long COVID

**DOI:** 10.1099/jgv.0.002172

**Published:** 2025-12-01

**Authors:** Marina Metaxaki, Ranjana Ram, Marianne Perera, Mark Wills, Benjamin A. Krishna, Nyarie Sithole

**Affiliations:** 1 Cambridge Institute of Therapeutic Immunology & Infectious Disease (CITIID), Cambridge CB2 0AW, UK; 2 Department of Medicine, University of Cambridge, Cambridge CB2 0QQ, UK; 3 Department of Infectious Diseases, Cambridge University Hospitals NHS Foundation Trust, Cambridge CB2 0QQ, UK

**Keywords:** antibody, long COVID, longitudinal, T cell

## Abstract

After severe acute respiratory syndrome coronavirus 2 (SARS-CoV-2) infection, a minority of patients experience persistent or emerging symptoms, termed ‘long coronavirus disease (COVID)’ or post-acute sequelae of COVID-19. The molecular causes of long COVID remain unclear, but disrupted immune functions, such as inflammation and immune deficit, have been posited as factors. In this retrospective cohort study, we measured markers of immune function in a group of patients with long COVID up to 40 months post infection. As proxies for immune function, we measured serum antibody levels, antibody neutralizing capability and production of IFN gamma (IFN-*γ*) and IL-2 against SARS-CoV-2 and other viral peptides. As expected, serum antibody levels increased over time with vaccinations and reinfections with later variants of SARS-CoV-2. Patients also showed corresponding increasing SARS-CoV-2-specific IL-2 responses and stable IFN-*γ* responses. We observed no significant differences in immune responses among patients with ongoing long COVID, those who had recovered from it or individuals who recovered from acute COVID-19. Overall, we found no indication of a reduction in these aspects of immune function after SARS-CoV-2 infection. This study provides a valuable foundation for further research aimed at understanding the causes of long COVID.

## Data Availability

Anonymized data is available upon request.

## Introduction

Long coronavirus disease (COVID), alternatively referred to as PASC- post-acute sequelae of coronavirus disease 2019 (COVID-19) or post-COVID condition (PCC), is characterized by persistent or emerging symptoms beyond the acute phase of COVID-19 [1]. The diverse and fluctuating symptom profile likely points to long COVID being a multi-syndromic condition rather than a single entity [2]. Furthermore, there is currently a lack of consensus on the causes, symptom profile, diagnostics or treatments for Long COVID [2,3]. With so many people having been infected with SARS-CoV-2, and unclear benefits of COVID vaccination on PCCs [4,5], long COVID could constitute an ongoing public health and economic burden. PCCs/complications include, but are not limited to, fatigue, cardiovascular disorders, respiratory symptoms, both new onset and worsening of pre-existing heart diseases [6,7] and potentially immunodeficiency. Many changes to the immune system have been noted after COVID-19, including reduced CD19 expression on B cells [8], markers of T cell exhaustion in patients with severe acute illness [9] and markers of inflammation 8 months post-COVID-19 [10]. There are also cases of unusual post-COVID opportunistic infections [11,14]. These irregularities in the immune system have been proposed as a cause of long COVID, along with autoimmunity, persistent SARS-CoV-2 infection, herpesvirus reactivation, coagulopathy and persistent organ damage caused by acute infection [2].

While the respiratory and systemic manifestations of COVID-19 have been extensively studied, the specific impact of long COVID on immune responses is yet to be fully understood. It is hypothesized that COVID-19 may induce an immunodeficient state which has implications for the study of long COVID. Indeed, those with long COVID have larger anti-spike IgG responses after vaccination [15] but also show signs of herpesvirus reactivations, probably indicative of immunodeficiency [13,14, 16,20]. We recently demonstrated in a cohort of patients with long COVID that T cell responses to SARS-CoV-2 antigens: spike (S), nucleocapsid (Nc) and membrane (M) proteins remain strong at least 6 months post-infection [21,22]. By recalling the same patients over up to 40 months, we were able to measure longitudinal serum antibody levels and IL-2 and IFN-*γ* over the years to assess if patients with long COVID show serological or cellular changes in immune function after COVID-19. During the course of this study, patients were vaccinated for SARS-CoV-2 and some were reinfected with later variants of the virus; these natural stimuli allowed us to assess immune responses post-COVID-19.

We focused on two fundamental aspects of the immune system: antibodies and cytokine production. We measured serum antibody levels specific to the S and Nc proteins of SARS-CoV-2 as well as their neutralizing capability. We also measured production of two cytokines, IL-2 and IFN-gamma (IFN-*γ*) following S, Nc and M peptide stimulation. Although it can be produced by other cell types, IL-2 is produced primarily by CD4^+^ T cells and plays a crucial role in regulating immune responses, particularly T cell proliferation and differentiation [23], while IFN-*γ*, produced by many immune cells including CD8^+^ cytotoxic T cells, is involved in antiviral defence and immune regulation [24]. These two cytokines can therefore act as a proxy for CD4^+^ and CD8^+^ T cell function.

To contextualize SARS-CoV-2-specific responses, we also stimulated cells with peptides from cytomegalovirus, Epstein–Barr virus and influenza (CEF) to assess the cytokine responses to other viruses. These data together allowed us to delineate responses to SARS-CoV-2 reinfections, spike-based COVID-19 vaccinations from 2021 to 2023 and memory to other viruses. Here, we show that IFN-*γ* responses remain stable over time, while serum antibody levels and IL-2 responses to SARS-CoV-2 tended to increase, likely due to immune boosting from vaccinations against, and reinfections with, SARS-CoV-2. Overall, we found an increase in anti-SARS-CoV-2 serum antibody levels over time and no decrease in IL-2- or IFN-*γ*-producing cells, which suggests maintenance of a robust functional immune system post-COVID-19 in patients with and without long COVID.

## Methods

### Acute and long COVID patient recruitment

The recruitment for this study commenced in April 2020. Across 40 months, 129 patients were recruited from the long COVID clinic at Addenbrooke’s Hospital who experienced ongoing symptoms for at least 3 months consistent with the definition of long COVID by NICE. Patients were triaged into this clinic after initial assessment by referring general practitioners, followed by an assessment of epidemiological and clinical history. Furthermore, patients were discussed in the long COVID multidisciplinary team meetings to ensure that they met the NICE criteria, and they were seen by all the appropriate medical teams including therapists where appropriate. Patients needed a confirmation of SARS-CoV-2 infection by a positive nucleic acid amplification test or a lateral flow test (taken at the time of infection). Patients were only included if no other medical explanation could be given for their symptoms (diagnosis by elimination). First, blood donations were taken at a median of 8.5 months post-primary infection. All patients were consented (according to the NIHR consenting protocol) during each appointment by signing the Adult NBR Joint ICF-PIS Vs9 03/08/2021 REC - 17/EE/0025 and the NIHR COVID-19 health questionnaire.

In March 2021, all consented individuals were contacted to follow up and 33, 17 and 13 of them attended a second, third and a fourth follow-up appointments, respectively. This corresponded to a median of 19, 28 and 39 months post primary infection, respectively. The follow-up appointments took place at the Clinical Investigation Ward (CIW) of the Addenbrooke’s Centre for Clinical Investigation (ACCI).

Acute 13 COVID patients were initially recruited by the NIHR BioResource Centre Cambridge through the ARIA study with ethical approval from the Cambridge Human Biology Research Ethics Committee (HBREC.2014.07) between November 2021 and January 2022. This cohort was infected between January and May 2022 with infection confirmed by either positive reverse transcription quantitative polymerase chain reaction (RT-qPCR) or lateral flow test.

### Sample collection and ethics

3×9 ml sodium citrate tubes were collected for FluoroSpot analysis, and an additional 1 ml was collected without anticoagulant for serum storage. To ensure anonymity, each patient was linked to a K number, and the data were stored in password-protected folders. Blood samples were handled in the laboratories on level 5 of Addenbrooke’s Hospital and processed on the same day in sterile conditions.

### Anti-spike and anti-nucleocapsid antibody quantification

Anti-spike and anti-nucleocapsid antibodies were measured using the SARS-CoV-2 IgG ELISA Kit (Enzo Life Sciences, ENZ-KIT190-0001) and LEGEND MAX™; SARS-CoV-2 Nucleocapsid kit following the manufacturer’s instructions. Serum from each donor was diluted 1/2,000 as suggested by the manufacturer and then added in duplicate to the plate. Anti-spike and anti-nucleocapsid antibodies were detected using biotinylated anti-human Ab detection antibody and avidin-HRP. Plates were then developed using tetramethylbenzidine reagent (77247, 77248 BioLegend) for 15 min at room temperature, and the reaction was stopped with 2M sulphuric acid. Absolute levels of both antibodies were quantified against the standard curve.

### Neutralization assays

Virus neutralization assays were run using HeLa cells expressing ACE2 cells (initially developed by Rogers *et al*. [25] and kindly gifted by Ravi Gupta) which could be infected with lentivirus pseudotyped with SARS-CoV-2 spike (developed by Schmidt *et al*. [26] but also gifted by Ravi Gupta), carrying the luciferase gene. Pseudotyped virus was incubated for 1 h at 37 °C with 1 : 3 serially diluted, heat-inactivated serum samples from the donors discussed, after which HeLa-ACE2 cells were added. Following a 48-h incubation, luciferase activity was measured using the BrightGlo Luciferase Assay System (Promega, UK). To control for background light levels, virus-only and cell-only controls were included and subtracted from all samples. Data were analysed in GraphPad Prism where 50% neutralization (ID50) values were calculated and the limit of detection for neutralization was set at an ID50 of 40. Within each group, the ID50 values were summarized as geometric mean titre.

### Peripheral blood mononuclear cell isolation for FluoroSpot analysis

Four citrate 9NC 0.106 Mol/9 ml bottles were collected and remained in these bottles until they reached the laboratory. After the blood was transferred into a 50 ml sterile falcon tube (~30ml of blood) that was 1 : 1 using sterile PBS. First, erythrocytes were removed by layering onto 15 ml of histopaque-1077 Hybri-Max, in Sepmate-50 blood separation tubes and centrifuged at 400 ***g*** for 10 min (room temperature, acceleration and deceleration at maximum).

The resulting top layer of plasma and peripheral blood mononuclear cells (PBMCs) was transferred into a sterile 50 ml tube and centrifuged at 2,000 r.p.m. for 5 min (room temperature, acceleration and deceleration at maximum). The PBMC pellet was then resuspended into a 50ml of sterile PBS (wash). The wash was repeated two more times. On the second wash, the resuspended pellet was centrifuged at 400 ***g*** for 10 min (room temperature, acceleration and deceleration at maximum). Lastly, PBMCs were resuspended in freezing media at 10^7^ cells per vial and frozen in liquid nitrogen.

FluoroSpot plates [FluoroSpot (Mabtech AB, Nacka Strand, Sweden)] were coated overnight with anti-human IFN-*γ* and IL-2 antibodies at 4 °C. Vials of frozen PBMCs were thawed in TexMACS (Miltenyi Biotech) media and then incubated for 1 h in benzonase to promote cell survival. Cells were counted and then plated in FluoroSpot plates at 2×10^5^ PBMCs per well in TexMACS supplemented with 5% human AB serum (Sigma-Aldrich). A small aliquot of cells was retained for counting using a flow cytometer (below). PBMCs were incubated with ORF mix peptides (final peptide concentration 2 μg ml^−1^/peptide) covering the entirety of spike, membrane and nucleocapsid ORFs from the prototype (Wuhan) variant of SARS-CoV-2 in duplicate wells. Negative controls were included (TexMACS-only) and a positive control was a cocktail of anti-CD3 (Mabtech AB; RRID:AB_907218), *Staphylococcus* enterotoxin B (SEB) (Sigma-Aldrich) and phytohaemagglutinin (PHA) (Sigma-Aldrich). Plates were then incubated at 37 °C in 5% CO_2_ for 48 h. After this, FluoroSpot plates were developed using the manufacturer’s instructions. Developed plates were read using an AID iSpot reader (Oxford Biosystems, Oxford, UK) and counted using AID EliSpot v7 software (Autoimmun Diagnostika GmbH, Strasberg, Germany) using distinct counting protocols for IFN-*γ* and IL-2 secretion. Data from AID EliSpot v7 software was then quality controlled to remove any false spots (Fig. S1A, available in the online Supplementary Material), and the number of spots in the negative control wells was subtracted from the stimulated wells to remove background. Final spot values were then divided by the number of cells plated (as counted by flow cytometry below) to produce spot forming units (SFU) per million PBMCs for each condition.

### Flow cytometry quantification of cells

To objectively measure the number of cells plated for each donor, after thawing PBMCs, an aliquot was stained with 2 μl each of live/dead dye for 15 min at 4 °C. Exactly 90 μl of the aliquot was run on an Accuri flow cytometer and live cells were quantified based on live/dead staining (Fig. S1B).

## Results

### Long COVID study cohort’s longitudinal clinical profile

A total of 129 patients were recruited from the long COVID clinic at Addenbrooke’s Hospital, Cambridge, UK. These patients were clinically assessed for long COVID symptoms and in all cases determined to have symptoms consistent with long COVID which also significantly impaired their quality of life. Of these, 33 returned at least once for follow-up blood donation, and 13 returned for 4 donations spread across a period of 40 months. The most common reason for the low turnout beyond the second donation was long COVID symptom resolution, although those with resolved long COVID symptoms were still encouraged to participate. Over the course of the study, the gender ratio moved from 64% female to 46% male, while the average age of participants remained stable (Table 1).

**Table 1.** Demographic and baseline characteristics of enrolled long COVID patients

Symptom	Long COVID	Long COVID	Long COVID	Long COVID	Pre-acute COVID	Post-acute COVID
Characteristic	Visit 1	Visit 2	Visit 3	Visit 4	Visit 1	Visit 2
Median date of bleeds	December 2020	January 2022	August 2022	July 2023	January 2022	November 2022
Number	33	33	15	13	13	13
% female (male)	64 (36)	64 (36)	53 (47)	46 (54)	54 (46)	54 (46)
Age (±SD)	45±14	45±14	47±9.5	47±9.5	39.6±12.5	40.3±12.5
Hospitalised during acute infection	3	3	0	0	n/a	0
Median time since symptom onset (months)	8.5	20	28	39	n/a	Resolved
Vaccinated (1 dose, 2 doses)	8 (3, 5)	33 (0, 33)	15 (0, 15)	13 (0, 13)	13 (0, 13)	13 (0, 13)
Confirmed reinfection (1 reinfection, 2 reinfections)	0 (0, 0)	12 (12, 0)	8 (6, 2)	10 (8, 2)	n/a	0

n/a - not applicable.

Eight participants were vaccinated before the first blood donation, and all participants received two vaccine doses between the first and second bleeds (Table 1). At first bleed, no participant reported having had more than one confirmed SARS-CoV-2 infection; however, reinfections occurred rapidly between bleeds 2 and 3, consistent with the Omicron wave (BA.1 and subsequent lineages) of SARS-CoV-2 in the UK [27]. By the final bleed, 10/13 participants had had multiple infections confirmed by RT-qPCR or lateral flow testing.

**Fig. 1.**
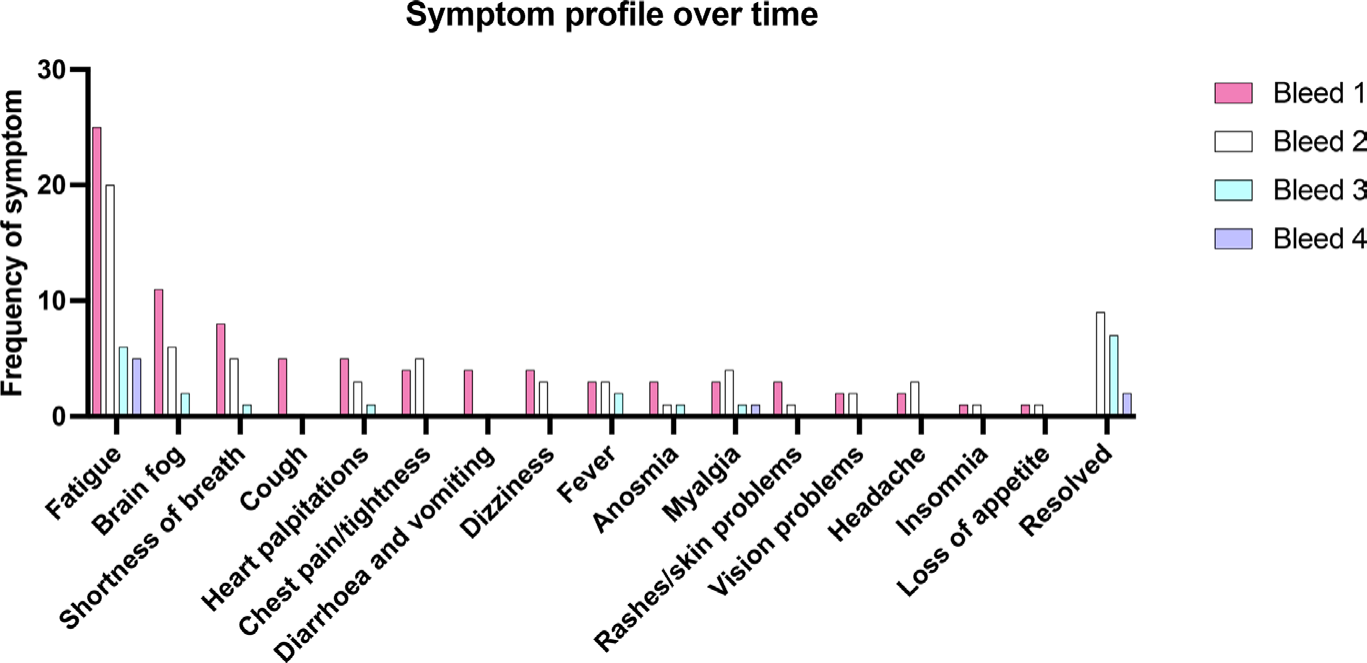
Patient-reported symptoms at each presentation. At each clinic visit, patients with long COVID were invited to report any symptoms they associated with their long COVID. Patients were allowed to report multiple symptoms, so total frequency of all symptoms is greater than the number of patients. If the patient reported resolution of symptoms, they were counted as ‘resolved’. At visit 1 (*n*=33), patients had experienced one confirmed infection. At visit 2 (*n*=33), patients had been vaccinated at least once and 12/33 experienced a confirmed reinfection. By visit 3 (*n*=15), patients had been vaccinated at least twice and 8/13 experienced a confirmed reinfection. By visit 3 (*n*=13) patients had been vaccinated at least three times and 10/13 experienced a confirmed reinfection.

Over the time course of the study, some symptoms tended to resolve faster than others, such as persistent cough, diarrhoea and vomiting, which resolved between visits 1 and 2. Later visits were dominated by fatigue and brain fog with some persistent myalgia and anosmia (Fig. 1).

A control cohort of 13 donors was recruited prior to SARS-CoV-2 infection and recalled 6 months post-infection and reported no ongoing symptoms 4 weeks post-infection. This group acted as both a negative control for uninfected immunity (visit 1) and a positive control for those without Long COVID (visit 2). This control cohort had a similar percentage of females (64%) and was ~5 years younger than the long COVID cohort (Table 1).

### Total antibody levels and antibody neutralizing capability continued to increase with multiple exposures from vaccination or infection

**Fig. 2.**
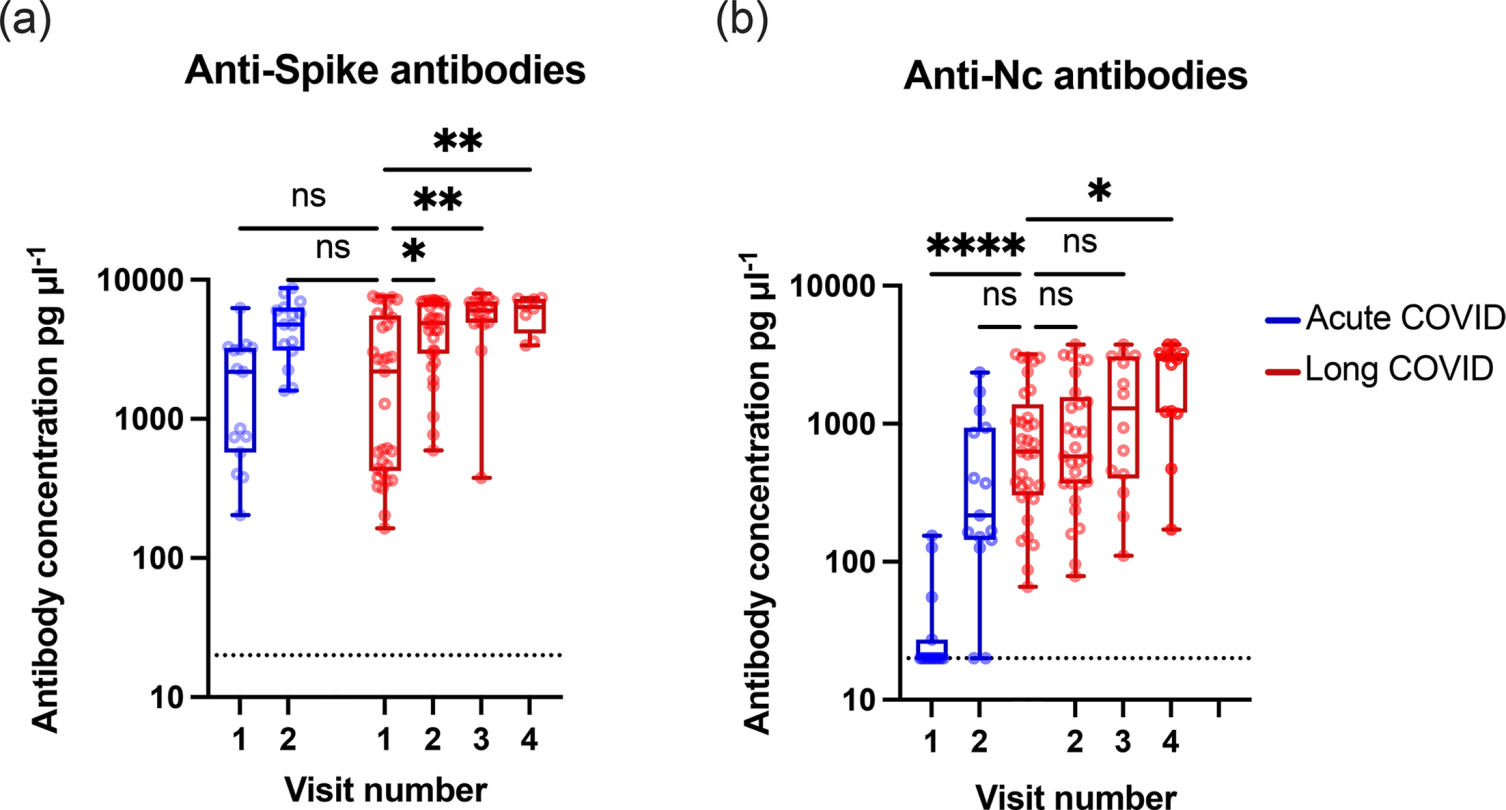
Antibody levels against spike rise with vaccination and rise with reinfections for nucleocapsid in both cohorts. Anti-S and anti-Nc antibodies were measured by ELISA from diluted serum. *P* values between visits were calculated using Kruskal–Wallis one-way ANOVA with Dunn’s multiple comparison test between the first visit for long COVID patients and all other visits. *P<0.05, **P<0.01. Each circle represents a single donor; changes in individual donors over time are shown in Fig. S2. L.O.D=limit of detectability, which was 20 pg µl^−1^. The same data is shown for individual donors in Fig. S2. For each visit, the number of donors (*n*), confirmed infections (*i*), confirmed reinfections (*r*) and vaccinations (*v*) was as follows. Acute visit 1 (*n*=13, *i*=0, *r*=0, *v*=13), acute visit 2 (*n*=13, *i*=13, *r*=0, *v*=13), long COVID visit 1 (*n*=33, *i*=33, *r*=0, *v*=8), long COVID visit 2 (*n*=33, *i*=33, *r*=0, *v*=33), long COVID visit 3 (*n*=15, *i*=15, *r*=8, *v*=15) and long COVID visit 4 (*n*=13, *i*=13, *r*=10, *v*=13).

We measured absolute serum antibody levels specific for spike and nucleocapsid proteins using LegendMax ELISA kits (Fig. 2a). Absolute levels of anti-S antibody were similar between acute and long COVID at visit 1. This is after two doses of vaccination for the acute cohort or one infection with SARS-CoV-2 for the long COVID cohort. Anti-S levels are similar for both at visit 2 as well; at this point, both cohorts had been vaccinated twice and infected with SARS-CoV-2. Anti-S antibody levels continued to rise in the long COVID cohort, consistent with reinfections with the delta and omicron waves of SARS-CoV-2 variants between visits 2 and 4 (Fig. 2a).

For anti-Nc antibody levels, the acute COVID cohort starts with negligible antibody levels (visit 1) which then rise after infection (visit 2) (Fig. 2b). The anti-Nc antibody levels are similar between the acute and long COVID cohorts after a single infection (visit 2 and visit 1 respectively), but the long COVID cohort shows a significant increase in anti-Nc antibody levels after reinfection with omicron (visit 4) (Fig. 2b).

**Fig. 3.**
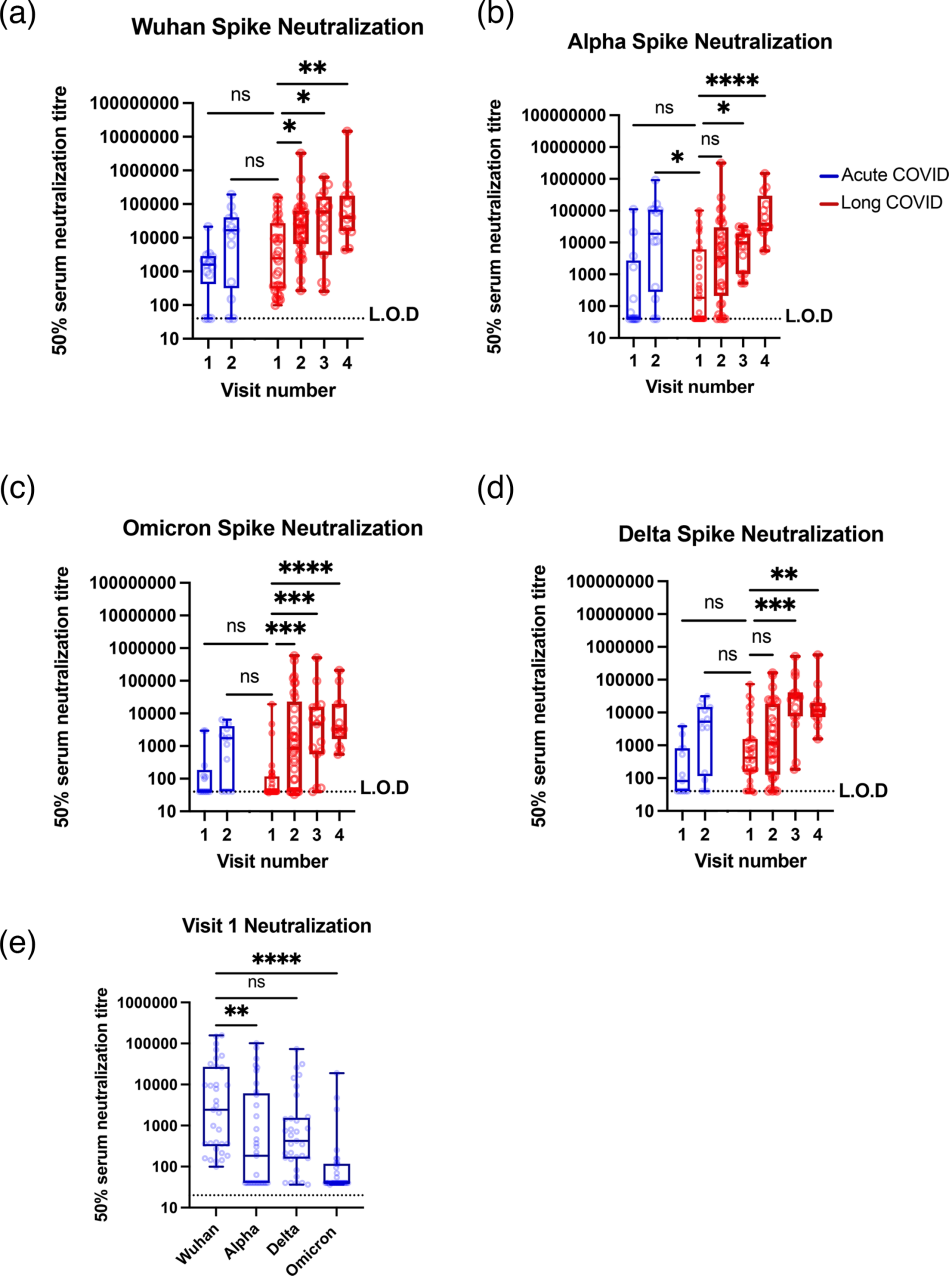
Antibody neutralizing titres against spike rise with vaccination and reinfections in both cohorts. Neutralization titres (ID_50_) of sera were measured against lentivirus pseudotyped with prototype (D614G), alpha, delta and omicron spike protein for each visit. *P* values between visits were calculated using Kruskal–Wallis one-way ANOVA with Dunn’s multiple comparison test between the first visit for the long COVID patient and all other visits. ^∗^*P*<0.05, ^∗∗^*P*<0.01, ^∗∗∗^*P*<0.001, ^∗∗∗∗^*P*<0.0001. L.O.D=limit of detectability, which was 40. The same data is shown for individual donors in Fig. S3. For each visit, the number of donors (*n*), confirmed infections (*i*), confirmed reinfections (*r*) and vaccinations (*v*) was as follows. Acute visit 1 (*n*=13, *i*=0, *r*=0, *v*=13), acute visit 2 (*n*=13, *i*=13, *r*=0, *v*=13), long COVID visit 1 (*n*=33, *i*=33, *r*=0, *v*=8), long COVID visit 2 (*n*=33, *i*=33, *r*=0, *v*=33), long COVID visit 3 (*n*=15, *i*=15, *r*=8, *v*=15) and long COVID visit 4 (*n*=13, *i*=13, *r*=10, *v*=13).

To measure the neutralizing capability of antibodies from each donor, we generated pseudotyped lentiviruses, with spike protein for prototype (Wuhan D614G), alpha, delta and omicron variants of SARS-CoV-2, and used these to infect HeLa cells exogenously expressing ACE2. Consistent with results in Fig. 2, neutralization capability increased as each cohort was vaccinated or infected with SARS-CoV-2 and neutralization was comparable between visit 2 for both cohorts: after vaccination and a single SARS-CoV-2 infection (Figs 3 and S2). At first, neutralization was stronger towards prototype spike compared to alpha, delta or omicron, as vaccines were based on the prototype variant of spike and long COVID patients were infected with the prototype variant of SARS-CoV-2 (Fig. 3e). Over time, the neutralizing capability of other variants increased as long COVID patients were exposed to these variants. Interestingly, the acute COVID visit 1 cohort, who had only had two doses of SARS-CoV-2 vaccine, had relatively low neutralization capability against alpha, delta and omicron variants of SARS-CoV-2, which increased significantly after infection with omicron (Fig. 3e).

Taken together, these data suggest no difference in antibody levels or neutralizing capacity between those who recovered from acute COVID-19 and people experiencing long COVID.

### IL-2 responses increased over the study period while IFN gamma responses remained stable

**Fig. 4.**
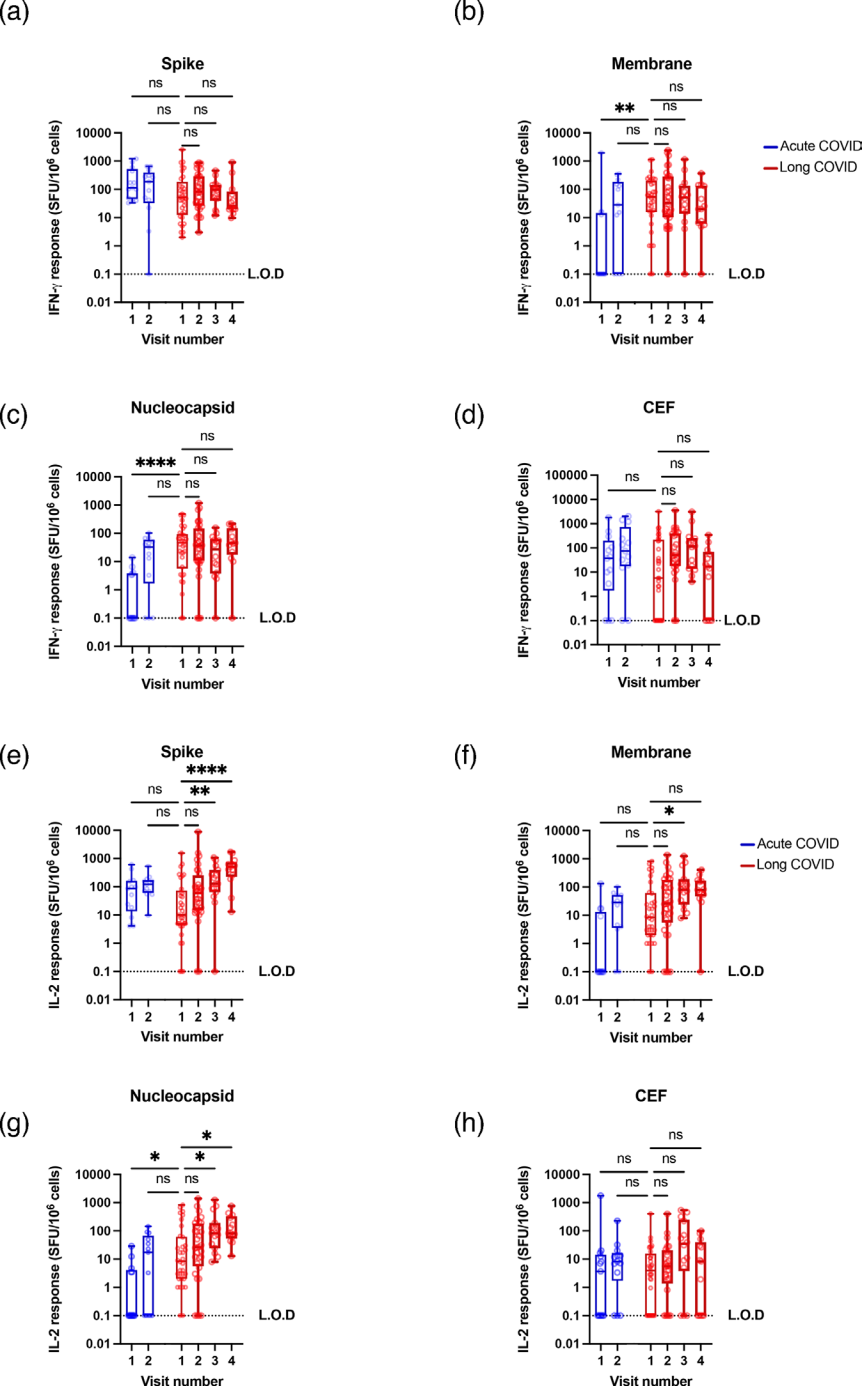
Antigen-specific IL-2 responses increase over time, while non-specific IL-2 responses and IFN-*γ* responses remain stable. PBMCs were isolated from long COVID patients 1–4 times after diagnosis of long COVID (red), or before and 6 months after acute COVID-19 (blue). These PBMCs were stimulated with spike (**a**), membrane (**b**), nucleocapsid (**c**) or CEF (**d**) peptides. IL-2 responses were measured by FluoroSpot assay as SFU per million PBMCs. IFN-*γ* responses were similarly measured by FluoroSpot for spike (**e**), membrane (**f**), nucleocapsid (**g**) or CEF (**h**). Each condition was run in duplicate and the number of spots was quantified against a peptide-negative, unstimulated control which was subtracted to remove background cytokine production. Zero results are set as 0.1 to allow their inclusion on a log scale. L.O.D.=limit of detection. Significance calculated by Kruskal–Wallis ANOVA, with Dunn’s multiple comparison test between the first visit for the long COVID patient and all other visits. The same data is shown for individual donors in Fig. S4. For each visit, the number of donors (*n*), confirmed infections (*i*), confirmed reinfections (*r*) and vaccinations (*v*) was as follows. Acute visit 1 (*n*=13, *i*=0, *r*=0, *v*=13), acute visit 2 (*n*=13, *i*=13, *r*=0, *v*=13), long COVID visit 1 (*n*=33, *i*=33, *r*=0, *v*=8), long COVID visit 2 (*n*=33, *i*=33, *r*=0, *v*=33), long COVID visit 3 (*n*=15, *i*=15, *r*=8, *v*=15), long COVID visit 4 (*n*=13, *i*=13, *r*=10, *v*=13).

We measured IL-2 production after antigen-specific stimulation with either S-, Nc-, M- or cytomegalovirus-, Epstein–Barr virus- and influenza (CEF)-specific peptide pools. These stimuli allowed us to distinguish between SARS-CoV-2 memory immune responses and general anti-viral immune responses. We also measured the overall capacity of patient T cells to respond using a positive control cocktail of anti-CD3, SEB and PHA to stimulate responses from all T cells. As expected, for the acute COVID cohort, Nc and M responses increase between visits 1 and 2 after donors were infected with SARS-CoV-2 (Fig. 4). Spike responses increased only marginally as patients had already been vaccinated with spike-based vaccines before visit 1, while CEF responses were stable (Fig. 4a, e).

In the long COVID cohort, this analysis revealed a statistically significant, gradual increase in S-, Nc- and M-specific T cell IL-2 responses over time (Figs 4 and S4A–D). The effect was strongest for spike-stimulated IL-2 responses, which increased 50-fold from 10 to 517 SFU per million PBMCs and weakest for membrane responses, rising from 8.5 to 80 SFU per million PBMCs. The responses to the CEF control remained stable over time, indicating that the increases in IL-2 production are specific for SARS-CoV-2. Similarly, overall T cell responses from the positive control cocktail were stable across cohorts over time, indicating that IL-2 production was specific to SARS-CoV-2 (Fig. S5).

We also performed the same analysis but measured IFN-*γ* production (Fig. 4e–h). As expected, responses to Nc and M increased significantly between visits 1 and 2 for the acute COVID cohort as they were infected for the first time with SARS-CoV-2. Otherwise, we found that the frequency of IFN-*γ*-producing T cells remained stable and there was no statistically significant change in either S-, Nc-, M- or CEF-specific IFN-*γ* responses over time (Figs 4 and S4E–H).

### Immune stability is similar regardless of reinfections

**Fig. 5.**
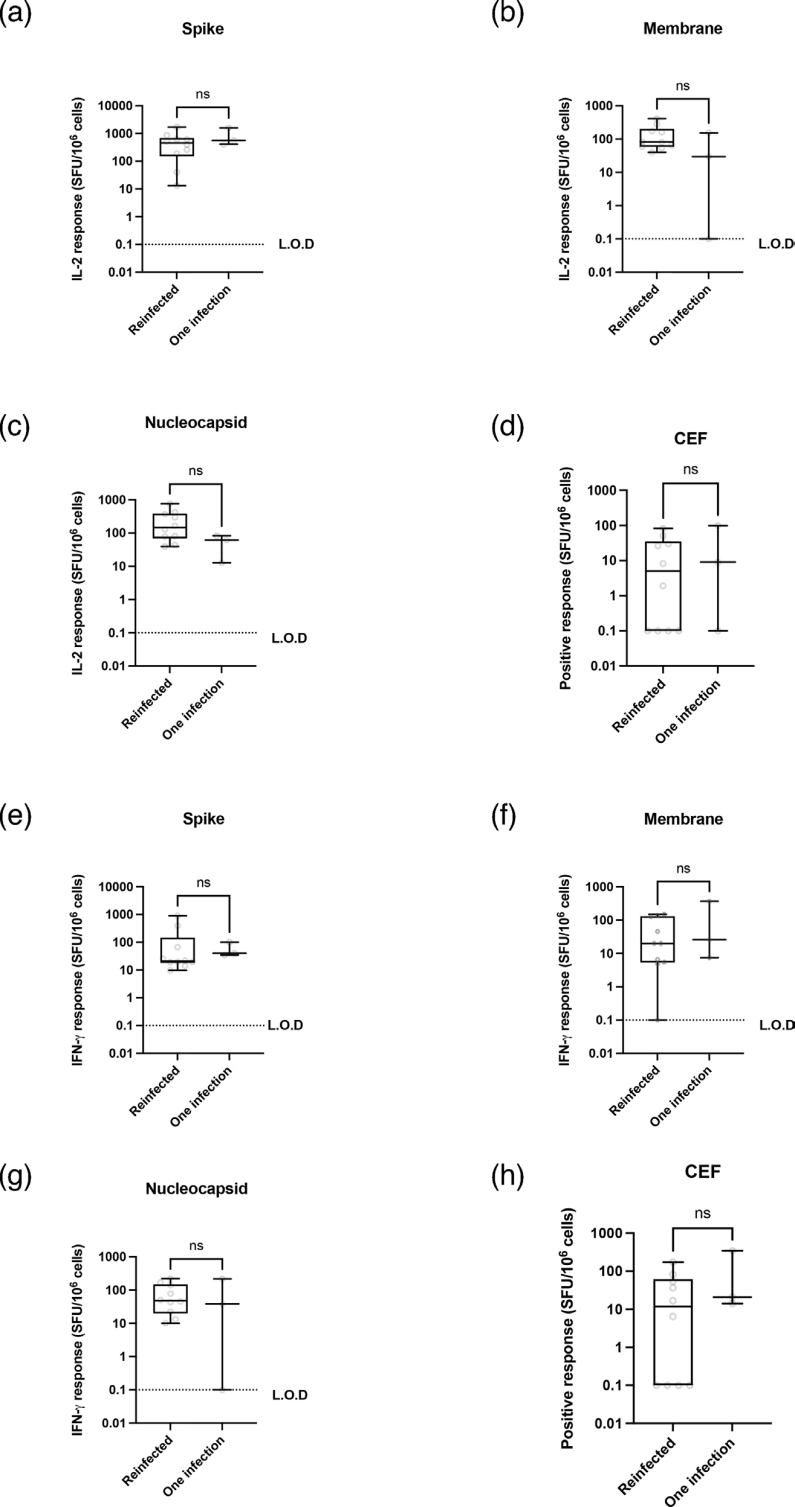
No statistical differences in cytokine responses between once-infected and reinfected donors. Donor cytokine responses (*n*=13, *r*=10, *i*=13) from Fig. 4 visit 4 were separated based on reported reinfections and plotted separately for comparison. Statistical analysis (Mann–Whitney U tests) showed no statistically significant results. L.O.D.=limit of detection.

Reinfection with SARS-CoV-2 could confound interpretations of our immunological data. To test this, we compared FluoroSpot data between long COVID patients who had reported a reinfection with SARS-CoV-2 and those who did not (Fig. 5). We found similar results for all cytokine responses, but did note a statistically insignificant reduction in the median IL-2 responses to membrane and nucleocapsid (Fig. 5).

### Resolution of long COVID symptoms did not correlate with any measured variables in this study

**Fig. 6.**
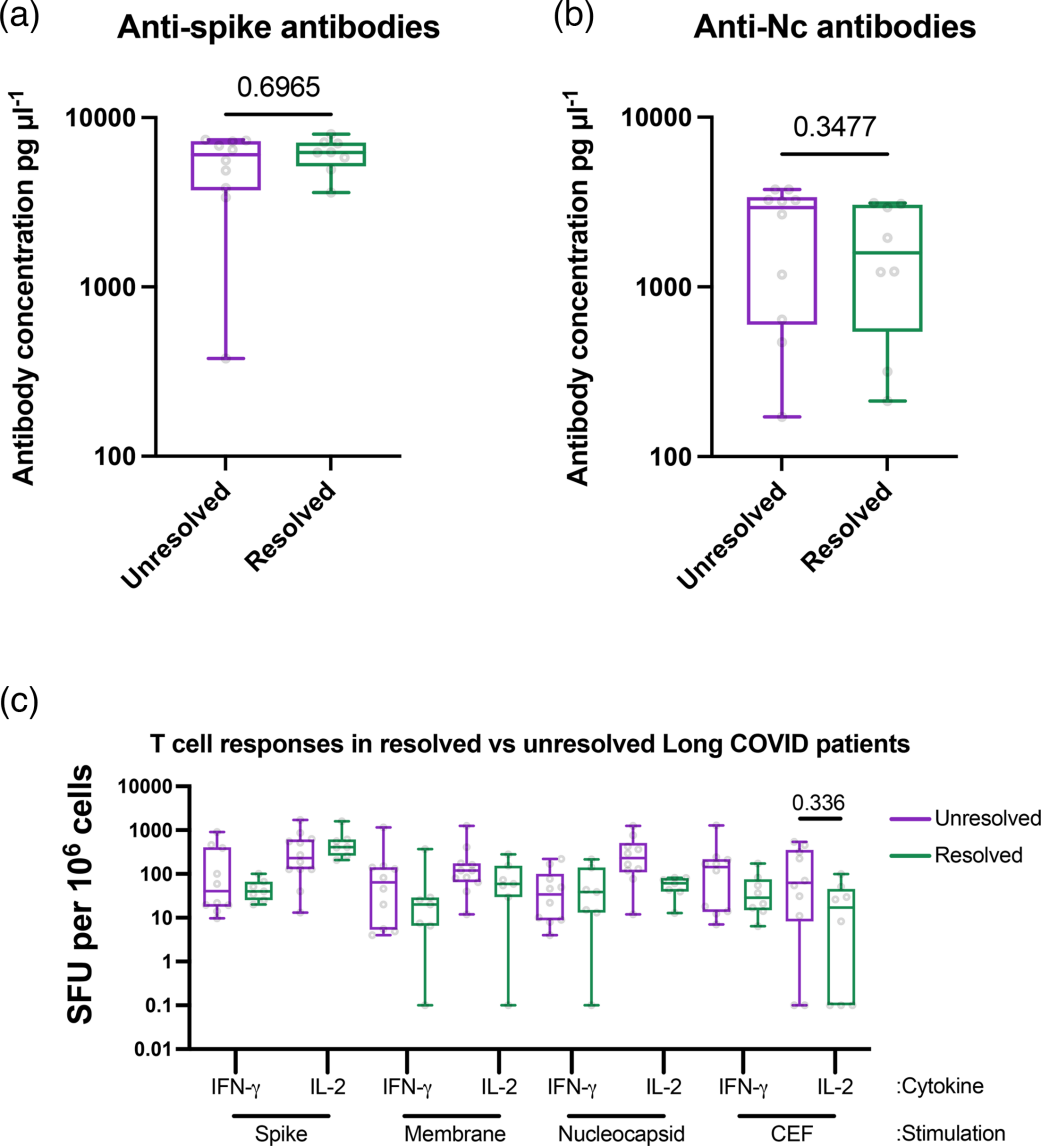
Long COVID symptom resolution does not correlate with serum antibody levels or T cell function. Data were compared between nine patients who reported continuing long COVID symptoms and eight patients who reported resolved long COVID symptoms. (a, b) Serum antibody levels from Fig. 2 were compared and significance was tested by Mann–Whitney U tests. (**c**) T cell responses from Figs3  4 were compared and significance was tested by Kruskal–Wallis ANOVA, with Dunn’s multiple comparison test between unresolved and resolved for each condition. No results were statistically significant. *P* values for each pairwise comparison were *P*>0.999 except where indicated.

Finally, of the long COVID patients who attended third and fourth visits, almost half (7/15 for visit 3 and 7/13 for visit 4) reported their long COVID symptoms had resolved before the visit. We therefore decided to test whether the patients whose symptoms had resolved showed different immunological profiles to those who had not recovered, as this might correlate long COVID symptoms with immunodeficiency. We therefore compared anti-S (Fig. 6a) and anti-Nc antibody (Fig. 6b) titres as well as T cell responses (Fig. 6c) to each peptide pool in nine patients who reported persistent long COVID symptoms and eight patients who reported complete symptom resolution. Across ten different variables, we found no statistically significant changes.

In summary, we have tracked anti-SARS-CoV-2 antibodies, antiviral IL-2 and IFN-*γ* T cell responses in long COVID over time. We conclude that long COVID patients do not show deterioration of these immune functions months or years after initial SARS-CoV-2 infection, and resolution of long COVID symptoms does not correlate with changes in these immune functions.

## Discussion

Long COVID is defined as the continuation or emergence of COVID-19 symptoms at least 3 months after SARS-CoV-2 infection and remains a serious public health threat. A lack of understanding of the molecular mechanistic basis and drivers of long COVID precludes developing effective treatment options. Many causes behind long COVID have been postulated. These include persistent/permanent organ damage from acute COVID-19 [28,32], persistent SARS-CoV-2 infection or antigens [33,39], autoimmunity [17,40, 41], vasculopathy [42,45], herpesvirus reactivations [16,17, 19], inflammation [46,48] and immunodeficiencies [8,12]. Indeed, these mechanisms are not mutually exclusive and may be responsible for different associated long COVID symptoms, may cause or exacerbate each other and may be responsible for symptoms at different times after infection.

Some have suggested that SARS-CoV-2 may cause immunodeficiency in the months or years after acute infection [8,12], which, if so, would potentially lead to serious recurrent infections and to increased morbidity and mortality. Given this possibility and importance, we tested the longitudinal magnitude of anti-SARS-CoV-2 and other virus-specific immune responses from a group of patients diagnosed with long COVID across a 40-month time frame to assess the stability of both humoral and T cell-mediated immune responses. We also related this data to vaccinations against SARS-CoV-2 and reinfections with later viral variants as well as changes in symptom profiles over this timeframe.

It should be noted that serum antibody levels and IL-2 and IFN-*γ* responses to SARS-CoV-2 peptide stimulation are only one aspect of the immune response which we use here as a proxy for general immunity. Additionally, although we recruited a large number of long COVID patients at the start of the study, only 15 participated for the entire 40-month duration of the study. Indeed, it is possible that the patients who chose to return for the entire duration were in better health than those who dropped out, leading to a skewing in our data. This effect would be stronger at the later time points, due to the limited sample availability. To address these limitations, it is important that future studies have larger cohorts and evaluate immune responses in more depth, such as analysing Natural Killer (NK) cell, γδT cell and other cell functions in the future. Additionally, IL-2 and IFN-*γ* are not produced exclusively by CD4 and CD8+ T cells, respectively, and our data do not rule out the possibility that these cytokines are made by other immune cells. IL-2 is produced primarily by CD4+ T cells, but small amounts are also made by CD8+ T cells, NK and dendritic cells [49]. IFN-*γ* is produced by a large range of immune cells including innate immune cells. In particular, post-COVID immunodeficiency may be a rare syndrome which would not be detected in a smaller-scale study. Finally, as previously reported [46], patients with long COVID spontaneously produced IFN-γ, which was subtracted from analysis in this study, but could confound some analyses.

We found that almost half of the patients who returned for third and fourth visits recovered fully from their long COVID symptoms. However, of note is that certain symptoms, such as fatigue, persisted for much longer than others such as coughing (Fig. 1). This agrees with other studies which have found that neuropsychiatric symptoms also last longer than respiratory symptoms [7,50, 51]. It is also in keeping with long-term studies post-Middle East respiratory syndrome (MERS) and Severe Acute Respiratory Syndrome (SARS) where fatigue was noted to be a persistent feature [52].

Most of the long COVID cohort (10/13) were reinfected by visit 4. Studies understanding the effects of reinfection on COVID-19 symptoms tend to agree that the cumulative risk of PASC increases with reinfections, but that the risk of experiencing PASC after each subsequent reinfection decreases [53,56]. Some patients also report worsening of their long COVID symptoms after reinfection with SARS-CoV-2 [57]. Within our cohort, no patients reported new symptoms after reinfection with SARS-CoV-2. This difference with the literature may be due to differences in reporting – survey-based data might prompt a response from patients who would not volunteer the information while speaking to a clinician. Secondly, we did not ask specifically about the severity of symptoms, so symptom severity may have worsened without new symptoms emerging.

We found in the long COVID cohort that anti-S antibodies increased significantly between visits 1 and 2, corresponding to December 2020 and January 2022 (Fig. 2a), in line with the UK’s COVID-19 vaccination programme which gave at least two doses of vaccine to all willing members of the public over 18 years old. As vaccines in the UK were all based on adenovirus and mRNA delivery systems for the spike ORF, we would expect induction of anti-S but not anti-Nc responses. Anti-Nc antibodies (Fig. 2b) increased non-significantly between visits 2 and 3 (January 2022 and August 2022) and significantly between 2 and 4 (August 2022 and July 2023), which corresponded with the wave of infections with the omicron variant of SARS-CoV-2, which started around November 2021 [27]. These observations agree with the abundance of publications showing antibody responses to SARS-CoV-2 vaccines and infections [58,62]. The levels of anti-S and anti-Nc antibodies were very similar between acute and long COVID patients after vaccination and a single infection, suggesting that long COVID patients did not have an attenuated response to vaccination (Fig. 2). Similarly, neutralization assays showed that antibody neutralization capability was similar between acute and long COVID patients, suggesting affinity maturation is not affected in long COVID (Fig. 3). This is best noticed by comparing acute COVID visit 2 against long COVID visit 2, where both cohorts have been vaccinated and infected once. Together, these data are not consistent with other reports suggesting low or no SARS-CoV-2 antibody production, low baseline levels of IgG or low levels of receptor-binding domain and spike-specific memory B cells in long COVID patients [8,63, 64]. Indeed, our work agreed with a recent study suggesting that antibody levels against other coronaviruses increased after vaccination and infection with SARS-CoV-2 [65].

We found that the frequency of IL-2-producing, SARS-CoV-2-specific T cells gradually increased over time (Fig. 4e–h) regardless of the progression of long COVID symptoms (Fig. 6), while IFN-*γ*-producing, SARS-CoV-2-specific T cells maintained a more constant rate over time (Fig. 4a–d). Comparing the two cohorts at visit 2, when both cohorts have been vaccinated and infected once, no differences in cytokine production can be seen. This suggests that vaccinations and reinfections, which occurred in most of our cohort, induce a growing IL-2-producing T cell population, likely driven by CD4^+^ T cells, but not cytotoxic CD8+ T cell responses. These results agree with a similar recent study which also saw relatively similar levels of anti-S and anti-Nc CD4+ and CD8+ T cells in patients with or without long COVID 24 months post-infection, as well as T cell expansion after reinfection or vaccination [66]. These results are in contrast with human immunodeficiency virus (HIV) infection, characterized by progressive failure to mount T cell responses, where IL-2 and IFN-*γ* responses decrease gradually in HIV-infected individuals [67]. Given that our long COVID cohort also shows stable responses to CMV/EBV/flu peptides, this suggests that long COVID patients do not experience progressive anti-viral immunodeficiency and that patients with diagnosed long COVID do not show a failure to mount antibody or T cell-based immune responses. It should be noted that the CEF peptide panel is better optimized for HLA-I presentation, which tends to stimulate CD8+ T cells and IFN-*γ* responses. An alternative peptide pool optimized for HLA-II presentation, perhaps including a wider range of peptides such as from tetanus toxoid, would be useful in future studies. Our own work on this same cohort previously reported a non-statistically significant reduction in CD3+, CD4+ and CD8+ T cells in patients with long COVID [46], which has been corroborated in some cases by others [68] which our results suggest is not functionally significant. Others have also reported lower IFN-*γ* production against nucleocapsid peptide stimulation in patients with long COVID, which we do not see in this cohort [69].

In summary, we followed a cohort of patients diagnosed with long COVID with the primary objective to establish whether there was any evidence of deteriorating immune responses over time. We found that most patients with long COVID gradually recovered from their symptoms over time, but fatigue persists in a small proportion of patients. Patients maintained antibodies and T cell responses over time and specifically produced more antibodies and IL-2-producing T cells in response to vaccinations and reinfections. Our findings demonstrate that long COVID patients maintain a robust functional immune status with no evidence of immune deficiency based on clinical symptomatology and immune molecular assessment.

## Supplementary material

10.1099/jgv.0.002172 Uncited Supplementary Material 1.

## Abbreviations

COVID: coronavirus disease
COVID-19: coronavirus disease 2019
HIV: human immunodeficiency virus
NK cell: natural killer cell
PASC: post-acute sequelae of COVID-19
PBMC: peripheral blood mononuclear cells
PCC: post-COVID condition
PHA: phytohaemagglutinin
RT-qPCR: reverse transcription quantitative polymerase chain reaction
SARS: severe acute respiratory syndrome
SARS-CoV-2: severe acute respiratory syndrome coronavirus 2
SEB: *Staphylococcus* enterotoxin B
SFU: spot forming units
