# Supplementary material for: Robust antibody and T cell responses tracked longitudinally in patients with long COVID
Source: J Gen Virol. 2025 Dec 1;106(12):002172. doi: 10.1099/jgv.0.002172 (PMC12668617; doi:10.1099/jgv.0.002172)
Supplement: Uncited Supplementary Material 1. [file jgv-106-02172-s001.pdf]

Supplementary material

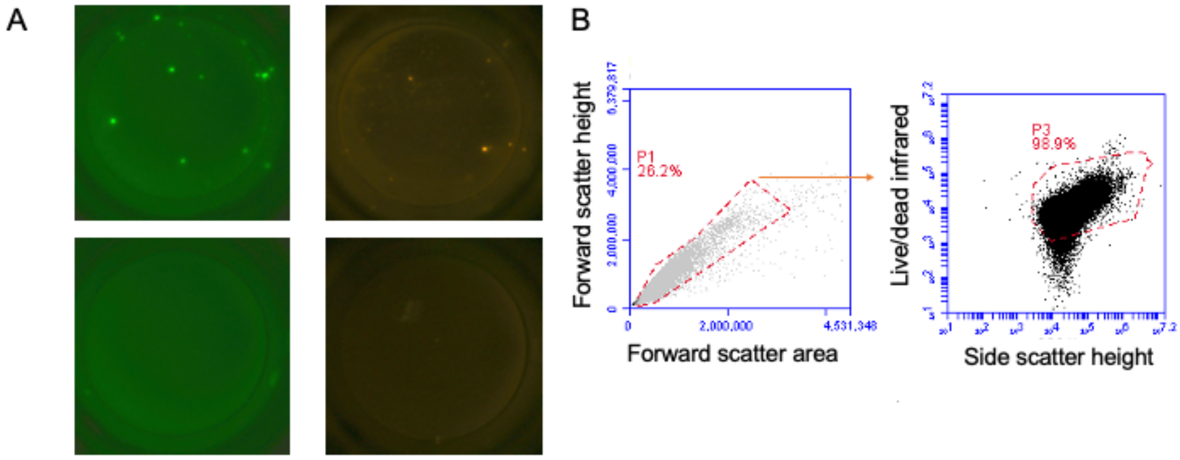

Figure S1: Representation of experimental procedure. (A) Representative images of wells of unstimulated PBMCs from a donor who showed no IFN-γ (green, left) or IL-2 (red, right) responses (bottom wells) and a donor which showed both (top wells), taken from the same plate with the same exposures. (B) Representative dot plots from 1 donor are shown illustrating the gating strategy for calculating the number of PBMCs loaded per well. In short, PBMCs were stained for survival using Live/Dead Far red dye. PBMCs were selected based on forward scatter height and area (left) and cells within this gate were then analysed for Live/Dead dye uptake, and those within gate P3 were counted as live PBMCs.

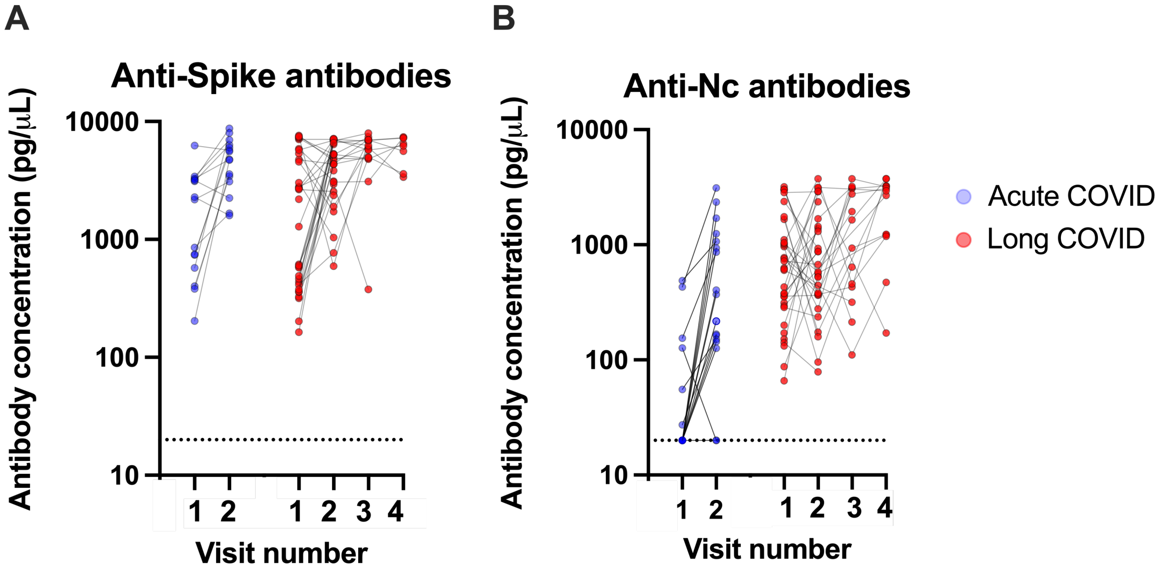

Figure S2: Data from figure 2 plotted to show donor changes over time. Anti-S and anti-Nc antibodies were measured by ELISA from diluted serum. Each circle represents a single donor which are shown here between visits.

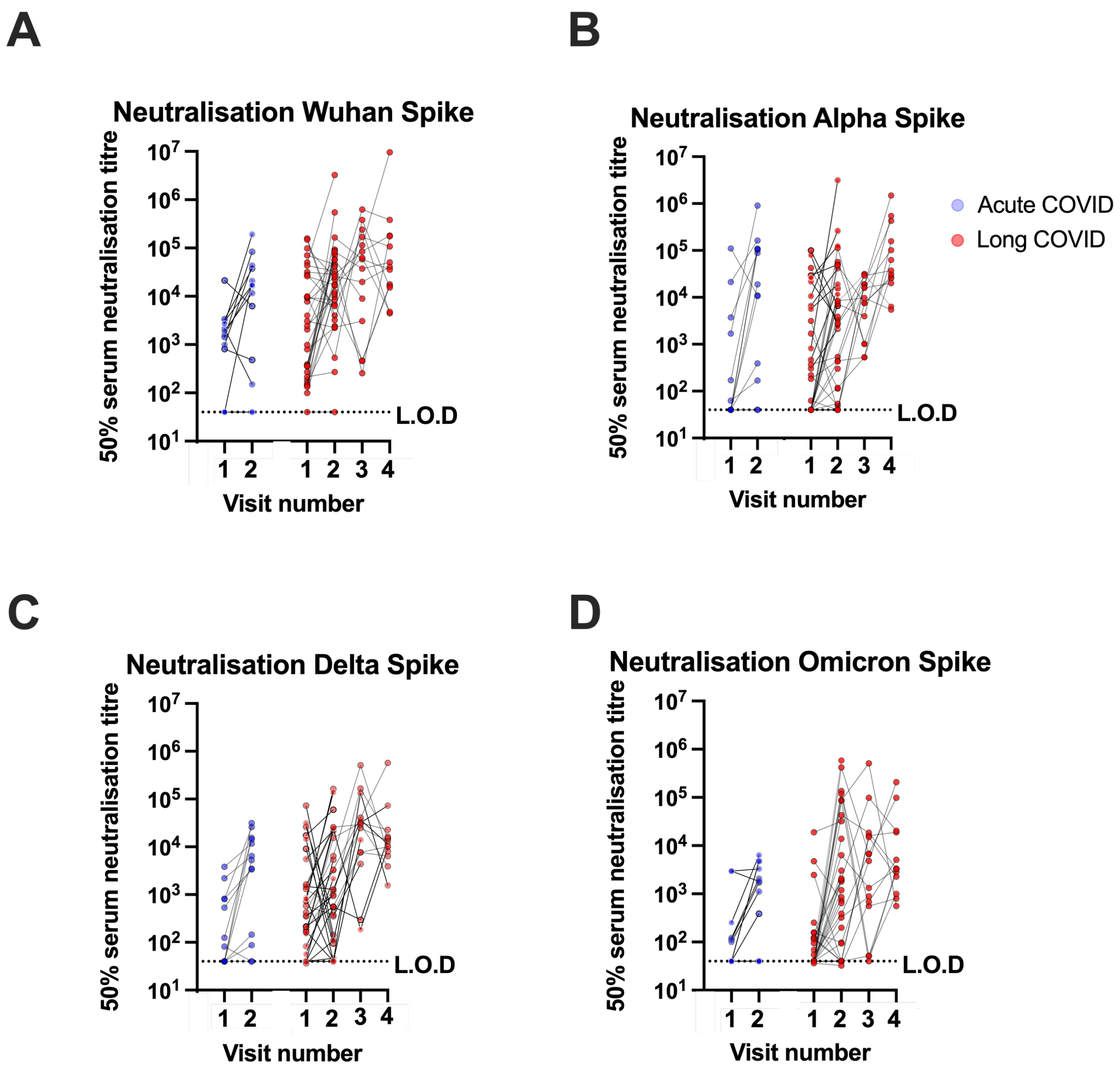

Figure S3: Data from figure 3 plotted to show individual donor changes over time. Neutralising antibody titres were measured by neutralising pseudotyped lentivirus with diluted serum. Each circle represents a single donor which are shown here between visits.

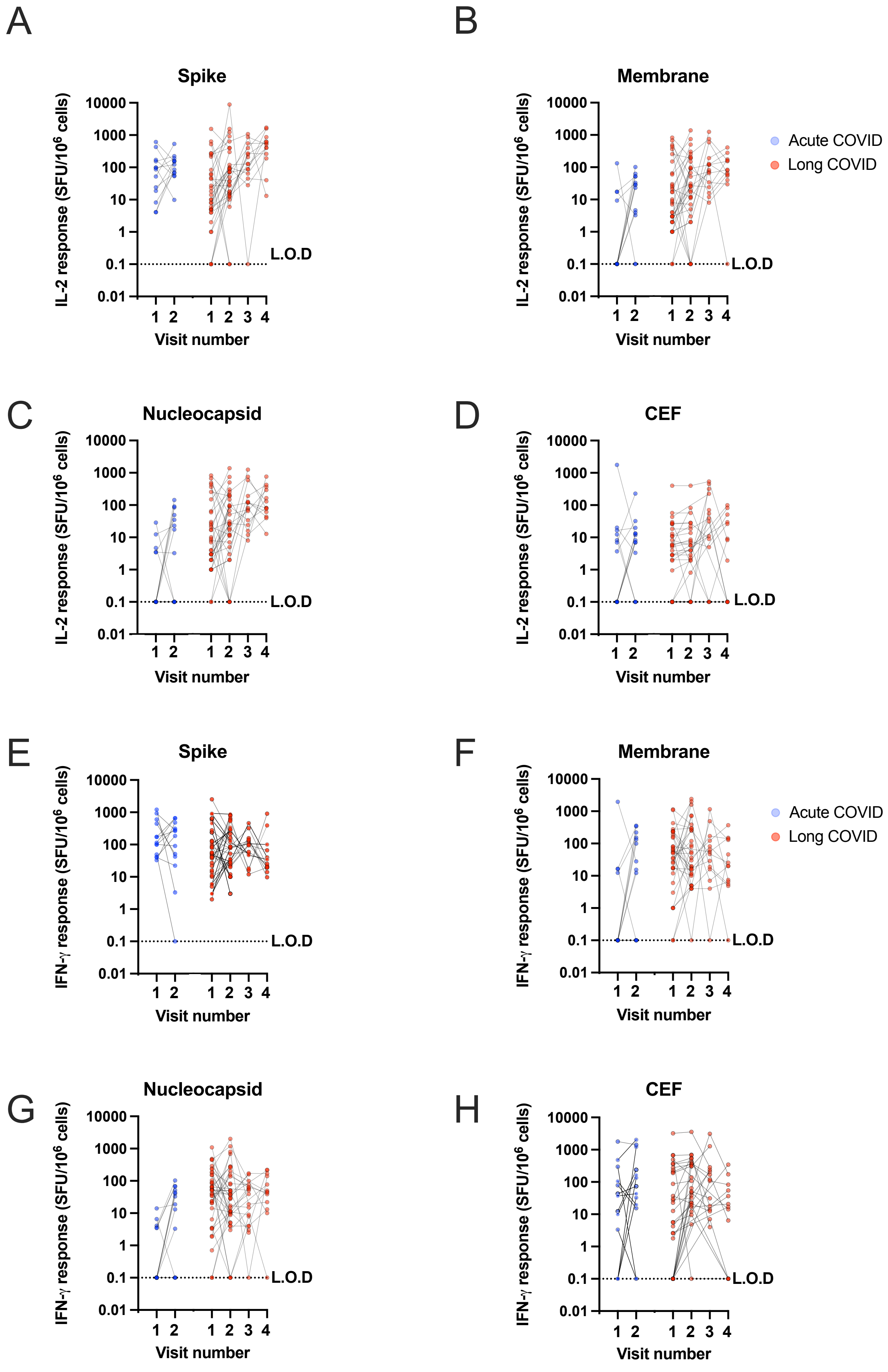

A
Spike
IL-2 response (SFU/10^6 cells)
10000
1000
100
10
1
0.1
0.01
L.O.D
1 2 1 2 3 4
Visit number
B
Membrane
IL-2 response (SFU/10^6 cells)
L.O.D
Visit number
Acute COVID
Long COVID
C
Nucleocapsid
IL-2 response (SFU/10^6 cells)
L.O.D
Visit number
D
CEF
IL-2 response (SFU/10^6 cells)
L.O.D
Visit number
E
Spike
IFN-γ response (SFU/10^6 cells)
L.O.D
Visit number
F
Membrane
IFN-γ response (SFU/10^6 cells)
L.O.D
Visit number
Acute COVID
Long COVID
G
Nucleocapsid
IFN-γ response (SFU/10^6 cells)
L.O.D
Visit number
H
CEF
IFN-γ response (SFU/10^6 cells)
L.O.D
Visit number

Figure S4: Data from figure 4 plotted to show individual donor changes over time. IL-2 and INF-γ responses to spike, membrane and nucleocapsid peptide pools as measured by FluoroSpot analysis are plotted to show donor changes between visits. L.O.D = limit of detectability.

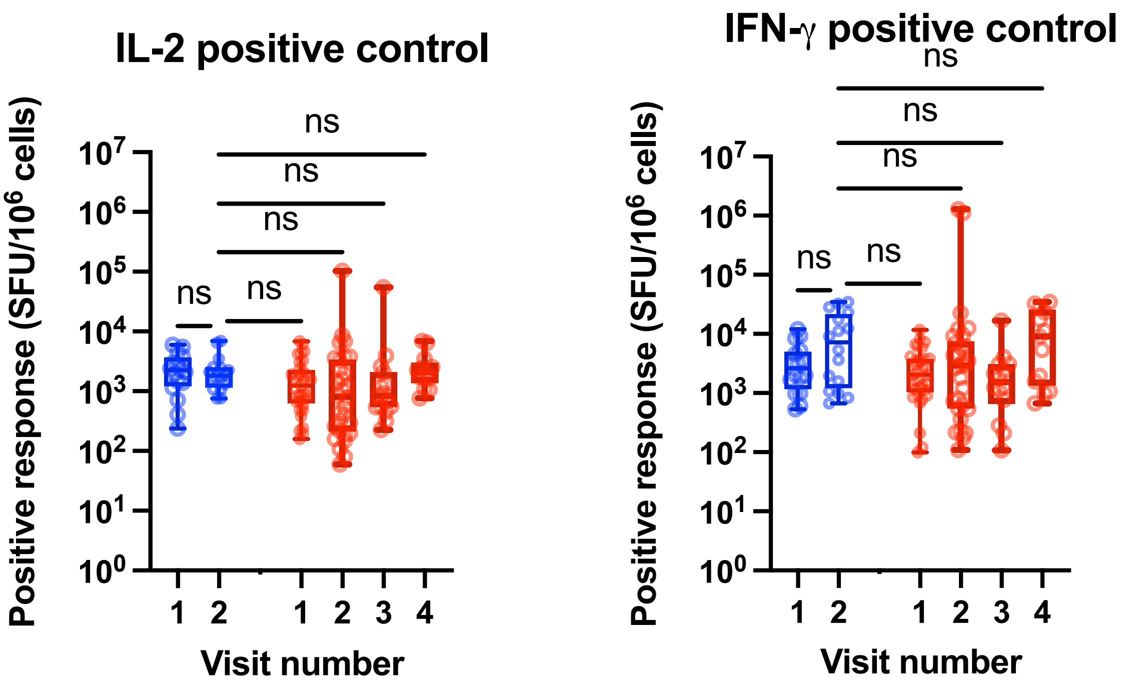

Figure S5: Overall T cell responsiveness does not change over time. PBMCs were isolated from Long COVID patients 1-4 times after diagnosis of Long COVID (red), or before and 6 months after acute COVID-19 (blue). These PBMCs were stimulated with a cocktail of anti-CD3, Staphylococcus Enterotoxin B and and phytohaemagglutinin, and IL-2 and INF-γ responses were measured by FluoroSpot analysis. Each condition was run in duplicate and the number of spots quantified against a peptide-negative, unstimulated control which was subtracted to remove background cytokine production. Significance calculated by Kruskal-Wallis ANOVA, with Dunn's multiple comparison test between first visit for Long COVID patient and all other visits. For each visit, the number of donors (n), confirmed infections (i), confirmed reinfections (r) and vaccinations (v) was as follows. Acute visit 1 (n=13, i=0, r=0, v=13), acute visit 2 (n=13, i=13, r=0, v=13), Long COVID visit 1 (n=33, i=33, r=0, v=8), Long COVID visit 2 (n=33, i=33, r=0, v=33), Long COVID visit 3 (n=15, i=15, r=8, v=15), Long COVID visit 4 (n=13, i=13, r=10, v=13).
